# Epidemiological investigation of sudden cardiac death in multiethnic Xinjiang Uyghur autonomous region in Northwest China

**DOI:** 10.1186/s12889-019-6435-8

**Published:** 2019-01-28

**Authors:** Jianghua Zhang, Xianhui Zhou, Qiang Xing, Yaodong Li, Ling Zhang, Qina Zhou, Yanmei Lu, Yinwen Fan, Abu Lizi, Wenhong Yan, Hongyan Wang, Meiling Zhai, Jianfu Bao, Liping Chen, Maihe Tuoti, Haidong Cui, Jian Wang, Baopeng Tang

**Affiliations:** 1 Department of Cardiology, First Affiliated Hospital of Xinjiang Medical University, 137 Liyushan South Road, Urmuqi, Xinjiang, 830011 China; 2 Xinhua Hospital, Ili, Xinjiang, Uygur Autonomous Region China; 3 Hotan People’s Hospital, Hotan, Xinjiang, Uygur Autonomous Region China; 4 Hami Center Hospital, Hami, Xinjiang, Uygur Autonomous Region China; 5 Bayingolin People’s Hospital, Bayingolin, Xinjiang, Uygur Autonomous Region China; 6 Xinyuan People’s Hospital, Xinyuan, Xinjiang, Uygur Autonomous Region China; 7 Barkol People’s Hospital, Barkol, Xinjiang, Uygur Autonomous Region China; 8 Yanqi People’s Hospital, Yanqi, Xinjiang, Uygur Autonomous Region China; 9 Lop People’s Hospital, Lop, Xinjiang, Uygur Autonomous Region China; 10 Aral People’s Hospital, Aral, Xinjiang, Uygur Autonomous Region China; 11 Wujiaqu People’s Hospital, Wujiaqu, Xinjiang, Uygur Autonomous Region China

**Keywords:** Sudden cardiac death, Ethnic group, Xinjiang

## Abstract

**Background:**

The epidemiological characteristics of sudden cardiac death (SCD) in the autonomous region of Xinjiang Uygur have been largely unknown. This study aimed to evaluate the incidence and demographic risk factors of SCD in Xinjiang, China.

**Methods:**

This retrospective study reviewed medical records from 11 regions in Xinjiang with different geography (north and south of the Tian Shan mountain range), gross domestic product, and ethnicity (Han, Uyghur, Kazakh, and Hui). SCD was defined as unexpected death due to cardiac reasons within 1 hour after the onset of acute symptoms, including sudden death, unexpected death, and nonviolent death. Monitoring was conducted throughout 2015. Demographic and mortality data were recorded and age-adjusted standardized risk ratio (SRR) was analyzed.

**Results:**

Among 3,224,103 residents, there were 13,308 all-cause deaths and 1244 events of SCD (784 men and 460 women; overall incidence 38.6 per 100,000 residents). SCD was associated with age (χ2 = 2105.3), but not geography. Men had an increased risk of SCD compared with women (SRR: 1.75, 95% CI: 1.10–2.79). The risk of SCD was highest in residents of the Uyghur (SRR: 1.59, 95% CI: 1.05–2.42) and Kazakh (SRR: 1.92, 95% CI: 1.29–2.87) compared with those of the Han. Poor economic development was associated with elevated risk of SCD (SRR: 1.55, 95% CI: 1.02–2.38).

**Conclusion:**

SCD is an important public health issue in China. Our understanding of the demographic differences on SCD in Xinjiang, China may improve the risk stratification and management to reduce the incidence and lethality of SCD.

## Background

Sudden cardiac death (SCD) is sudden, unexpected, natural death that occurs when the heart fails to contract [1]. SCD is a major public health issue worldwide. Coronary heart disease accounts for ~ 80% of SCD cases [2, 3], and is responsible for the greater portion of total deaths among adults in industrialized countries [4]. Across the world the incidence of SCD ranges widely, from 52.5 per 100,000 person-years in Asia to 111.9 per 100,000 person-years in Australia, as estimated by the numbers of out-of-hospital cardiac arrests attended by emergency medical services [5].

In China, Hua et al. [6]. conducted an epidemiological investigation of SCD that covered Beijing, Guangzhou, Yuxian, and Kelamayi. These locales are, respectively, the capital city of China in the north, a coastal metropolis of Southern China, a county in Northern China, and a city in the autonomous region of Xinjiang Uygur (hereafter referred to as Xinjiang) in Northwest China. The overall incidence of SCD during the year 2015 was 41.8 per 100,000, with specific rates for Beijing, Guangzhou, Yuxian, and Kelamayi of 49.9, 46.9, 39.3, and 29.3 per 100,000, respectively. These results showed that the incidence of SCD varied widely by geographic region.

Rates of SCD in China may also vary widely across a single geographic region. For example, while the study of Hua et al. [6]. sampled the single city of Kelamayi within Xinjiang, the data was far from representative of the entire region. The Tian Shan mountain range divides Xinjiang into two major areas, Southern Xinjiang and Northern Xinjiang, that differ in climate and demographics [7]. Xinjiang also borders many countries that influence the area culturally, and residing within are as many as 47 ethnic groups, including the Han, Uygur, Kazakh, and Hui. Each ethnic group varies by residence, culture, religion, genetics, and history.

We conducted an epidemiological investigation of 11 sites within Xinjiang to understand better the geographic, economic, and ethnic factors that may contribute to SCD in China.

## Methods

### Study population

The Ethics Committee of First Affiliated Hospital of Xinjiang Medical University, China approved the study. The study received all necessary administrative permissions to access and use the medical records.

To obtain a representative picture of SCD epidemiology across the entirety of Xinjiang, a 2-stage stratified cluster sampling method was applied in which the geographical distribution, socioeconomic development, and ethnic groups were weighed. We then analyzed the cases of SCD that occurred in 2015 within 11 chosen survey sites.

**Fig. 1:**
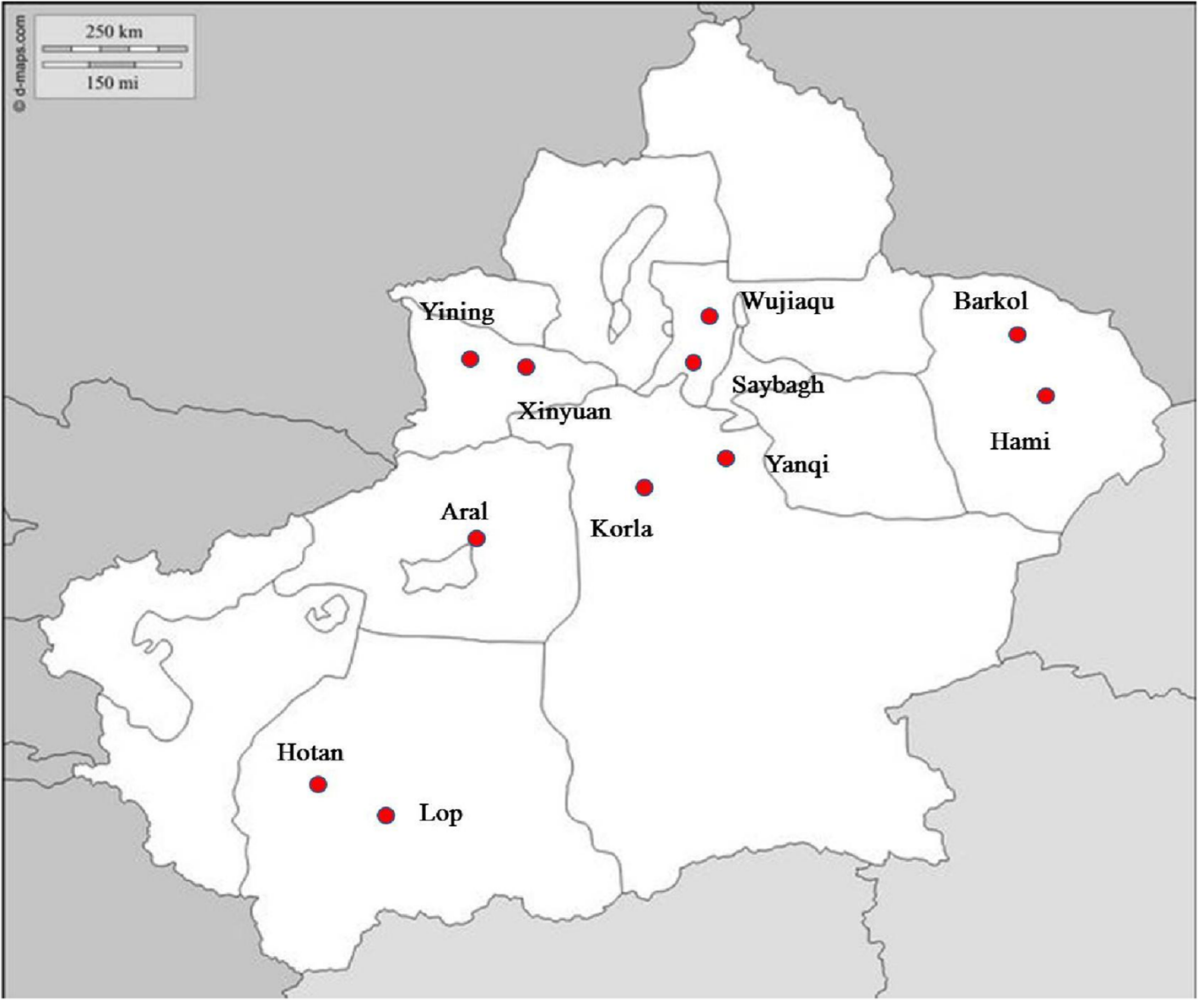
Geographic locations of 11 selected sites in Xinjiang. (The picture was referenced from http://d-maps.com/carte.php?num_car=22960&lang=en, and the 11 sites were marked using Photoshop software. This reference conforms to the company’s ordinance of free reference (http://d-maps.com/conditions.php?lang=en))

Specifically, in the first stage, 6 regions were selected from the 13 prefectural level regions (Ürümqi city; the prefectures Ili, Bayingolin, Hami, and Hotan; and Bingtuan), and in addition, the Xinjiang Production and Construction Corps (Bingtuan; Table 1). In the second stage, 2 city districts or counties were selected from each of the 6 regions (Fig. 1). Thus, 11 survey sites were finally selected, as follows: the Saybagh District of Ürümqi city; in Ili Prefecture, Yining city and Xinyuan County; within Bayingolin Prefecture, Korla city and Yanqi County; in Hami Prefecture, Hami city and Barkol County; within the Hotan Prefecture, Hotan city and Lop County; and of the Bingtuan, the cities of Aral and Wujiaqu.

**Table 1:** Eleven analyzed sites in Xinjiang Uyghur Autonomous Region in Northwest China by prefecture

	Site	Geographic area	Urban/Rural	GDP
Hami	Hami City	North	Urban	High
	Barkol County	North	Rural	High
Ürümqi City	Saybagh District	North	Urban	High
Ili	Yining City	North	Urban	Moderate
	Xinyuan County	North	Rural	Moderate
Hotan	Hotan City	South	Urban	Poor
	Lop County	South	Rural	Poor
Bayingolin	Korla	South	Urban	High
	Yanqi County	South	Rural	High
Bingtuan^a^	Wujiaqu	North	Rural	–
	Aral City	South	Rural	–

^a^Xinjiang Production and Construction Corps

For analysis, the 11 sites were categorized as northern or southern Xinjiang; urban or rural; and by per capita gross domestic product (GDP) classified as high, moderate, or poor (Table 1). Geographically, the selected sites in northern Xinjiang were: Ürümqi city, Ili Prefecture, Hami Prefecture, and Wujiaqu city. In southern Xinjiang, the sampling sites were: and Bayingolin Prefecture, Hotan Prefecture, and Aral city.

Sites were stratified as either urban or rural based on the locale of the majority of the population. The urban area sites were: Saybagh District, Korla city, Yining city, Hami city, and Hotan city. The rural area sites were: Xinyuan County, Barkol County, Yanqi County, Lop County, Aral city, and Wujiaqu city.

To detect the extent that economic factors influenced the age-adjusted death rate (ADR) due to SCD, the economic classification of the selected sites was based on per capita GDP in Renminbi (RMB), according to the 2014 Statistical Yearbook of Xinjiang Uygur Autonomous Region. Thus, sites with GDPs of 50,000, ~ 30,000, and < 10,000 RMB (that is, 7365, ~ 4419, and 1473 USD) were defined as economically high, moderate, and poor, respectively. The economically high, moderate, and poor sites were as follows: high, Ürümqi city, Bayingolin Prefecture and Hami Prefecture; moderate, Ili Prefecture and Bingtuan; and poor, Hotan Prefecture.

To investigate an association between SCD and age, residents were classified as ages < 18, 18–35, 35–60, or > 60 years. The mortality statistics of the following ethnic groups were also compared: Han, Uyghur, Kazakh, and Hui.

### Data collection

One local hospital at each selected site was designated as the coordinating local center for that site. Before the formal investigation, we signed a work protocol contract at each center. A senior physician from the cardiology department of the local center was assigned as the project leader for that center, and a junior physician served as an investigator. All project leaders and investigators were trained uniformly on project content and investigation techniques. At the provincial level, an expert panel consisting of 4 senior cardiovascular physicians was gathered for the final review of the SCD cases.

In China, certain family events are recorded in the household registration system, which serves as official recognition, from birth to death. Generally, when a family member dies, citizens are required to apply for permanent cancellation of household registration, which is necessary for the funeral and interment. Citizens provide the decedent’s death certificate, which includes name, date of death, and cause of death.

In the present study, in each quarter the local investigator gathered data from the administrative office that concerned heart-related deaths and filled in a death questionnaire, which included general information and disease information. If the death occurred in the hospital or a doctor was involved at an emergency scene outside the hospital, the investigator gathered the data from the hospital’s medical records to complete the death questionnaire. If the death occurred outside the hospital without doctor involvement, the relatives or witnesses of the decedent were interviewed to complete the death questionnaire.

The project leader of the local center was responsible for the first examination of the death questionnaires. Cases that did not conform to the SCD definition were excluded. The death questionnaires for cases of SCD were then delivered to the provincial institution for examination by an expert panel. Questionnaires with inadequate information for a confirmed diagnosis were returned to the local investigator for the required crucial information.

### SCD definition

SCD was defined as unexpected death due to cardiac reasons within 1 hour after the onset of acute symptoms [8–10], including sudden death, unexpected death, and nonviolent death. Acute symptoms included chest pain, loss of consciousness, respiratory arrest, or nausea [11]. Deaths during sleep, without a clear history of disease or illness or symptoms before falling asleep, were also described as SCD. In any situation, deaths were excluded that were due to obvious causes that were not cardiac-related.

### Quality control

To ensure that the study had complete data regarding SCDs, the local investigators were required to search for underreporting and instances of delayed household registration cancellations, by consulting neighborhood committee staff. In addition, at every quarter the death questionnaires were sent to a provincial center for disease control and prevention, to conduct a cross-comparison. Potential SCD cases that were suspected as missing were recorded and sent to a corresponding local investigator to check further.

### Statistical analysis

Statistical analyses were conducted using SPSS software version 22.0 (IBM, Chicago, IL, USA). Quantitative variables were described as mean ± standard deviation and qualitative variables as frequency.

To control for differences in age distribution and therefore increases in mortality due to aging, the SCD incidence rates of groups were age-adjusted using direct standardization prior to comparisons. Age comparisons among sites were conducted using variance analysis or the Kruskal-Wallis H test, depending on the homogeneity of variance. The standardized rate ratios (SRRs) were calculated to compare age-standardized SCD incidence rates among subgroups. Significant difference was counted if the 95% confidence interval of the SRR did not include the value of 1.0.

## Results

Mortality statistics that focused especially on overall deaths and SCDs were collected for the year 2015 at 11 sites in the region of Xinjiang in Northwest China (Table 2). The total population of residents was 3,224,103, and there were 13,308 all-cause deaths. Among these, there were 1244 cases of SCD (784 men and 460 women). Therefore the crude incidence rate of SCD was 38.58 per 100,000 residents.

**Table 2:** Demographics, mortality, and rates of SCD in 11 selected sites of Xinjiang in year 2015

Variable	Mortality, *n*			SCD, *n*			SCD mortality, %		SCD per 100,000	
	Subjects	All	Cardiac	Total	Men	Women	Per all	Per cardiac	CDR	ADR
Aral	36,183	122	31	14	10	4	11.5	45.2	38.7	24.7*
Barkol	105,540	571	157	41	22	19	7.2	26.1	38.9	37.3
Hami	214,176	1537	387	87	61	26	5.7	22.5	40.6	34.6
Hotan	346,586	1781	439	118	89	29	6.6	26.9	34.1	47.9
Korla	564,173	1573	395	203	129	74	12.9	51.4	36.0	36.2
Lop	270,237	1533	486	108	67	41	7.0	22.2	40.0	61.0*
Saybagh	549,899	1949	423	216	121	95	11.1	51.1	39.3	31.2
Wujiaqu	89,330	613	137	31	23	8	5.1	22.6	34.7	25.3
Xinyuan	325,590	1427	344	126	75	51	8.8	36.6	38.7	45.1
Yanqi	162,698	673	171	73	44	29	10.9	42.7	44.9	53.9
Yining	559,691	1529	492	227	143	84	14.9	46.1	40.6	43.8
Overall	3,224,103	13,308	3462	1244	784	460	9.4	35.9	38.6	38.6
*P*-value	–	–	–	–	–	–	< 0.001	< 0.001	0.827	< 0.001

*SCD* sudden cardiac death, *ADR* age-adjusted death rate, *CDR* crude death rate;

*P < 0.05

The overall mean age of the residents who suffered SCD was 67.5 ± 15.7 years, with significant differences among the 11 sampling sites (Table 2). The number of SCD cases exceeded 10% of all-cause deaths in Saybagh, Yining, Korla, Yanqi, and Aral. Overall, SCD accounted for 9.4% of all-cause deaths, and there were significant differences among the 11 sites.

The number of SCD cases among the total number of deaths due to cardiac disease ranged from 22.2 to 51.4% (Table 2). In Saybagh and Korla, these percentages were 51–52%, while that of Lop, Hami and Wujiaqu were 22–23%. The overall crude death rate due to SCD was 38.6 per 100,000 residents, ranging from 34.1 to 44.9, which was significantly different among sites. Concerning the ADR due to SCD, the highest was 61.0 in Lop and the lowest was 24.7 in Aral, a statistical difference between the two sites; across the 11 sites the ADRs due to SCD were comparable.

**Fig. 2:**
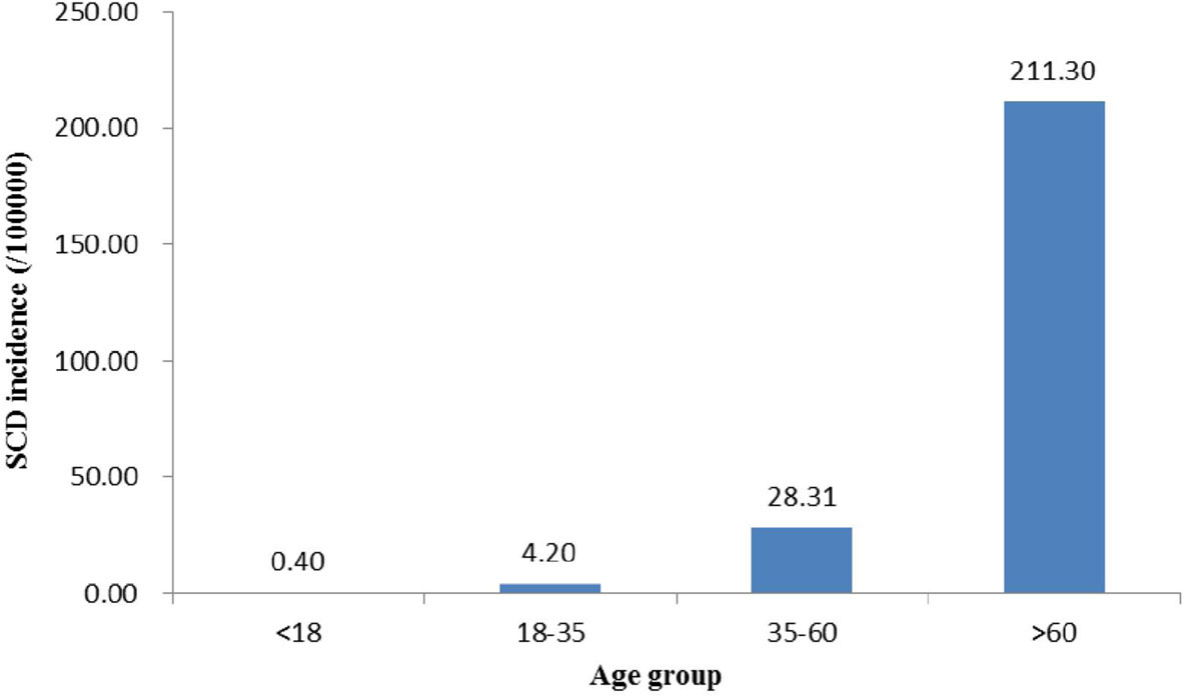
Age-adjusted SCD incidence among 11 sites in Xinjiang

When the residents were stratified by age, the incidences of SCD per 100,000 for the ages < 18, 18–35, 35–60, and > 60 years were 0.4, 4.2, 28.3, and 211.3, respectively (Fig. 2). The chi-squared test for trend indicated that rates of SCD increased with age, and the age groups differed significantly (χ2 = 2105.3, *P* < 0.05).

The ADR for SCD among men was 49.3 per 100,000 residents (95% CI: 46.8–51.7), which was higher than the 28.1 per 100,000 residents (95% CI: 26.3–30.0) for women. Men had an increased risk of SCD compared with women [SRR (95%CI)A: 1.75 (1.10–2.79)]. Among the four ethnic groups, comparing with the Han, the risk of SCD was similar [SRR (95%CI): 0.81 (0.50–1.31)] for the Hui while significantly increased risk of SCD was observed in the Uyghur [SRR (95%CI): 1.59 (1.05–2.42)] and the Kazakh [SRR (95%CI): 1.92 (1.29–2.87)] (Table 3).

**Table 3:** SCD among residents of 11 selected sites in Xinjiang

Variable	Subgroup	SCD incidence per 100,000				
		Subjects, *n*	SCD, *n*	Total	Age-adjusted	SRR (95% CI)
Gender	Female	1,589,527	460	28.9	28.1 (26.3–30.0)	1
	Male	1,654,576	784	47.4	49.3 (46.8–51.7)	1.75 (1.10–2.79)
Ethnic group	Han	1,582,354	635	40.1	36.3 (34.2–38.3)	1
	Uyghur	1,195,089	456	38.2	57.9 (55.2–60.5)	1.59 (1.05–2.42)
	Kazakh	230,115	83	36.1	69.9 (66.9–72.8)	1.92 (1.29–2.87)
	Hui	170,872	70	41.0	29.3 (27.4–31.2)	0.81 (0.50–1.31)
Area of Xinjiang	Northern	1,844,226	728	39.5	36.4 (34.3–38.5)	1
	Southern	1,379,877	516	37.4	42.4 (40.2–44.7)	1.16 (0.75–1.81)
Region	Rural	989,578	393	39.7	42.1 (39.9–44.4)	1
	Urban	2,234,525	851	38.1	37.3 (35.2–39.4)	1.13 (0.73–1.75)
Economic development	Highest	1,596,486	620	38.8	34.5 (32.5–36.6)	1
	Moderate	1,010,794	398	39.4	37.2 (35.1–39.3)	1.07 (0.68–1.71)
	Poor	616,823	226	36.6	53.5 (51.0–56.1)	1.55 (1.02–2.38)

*SCD* sudden cardiac death, *SRR* standardized rate ratio

Xinjiang is naturally divided geographically into the southern and northern regions. The ADR per 100,000 due to SCD in southern Xinjiang (42.4) was higher than that of northern Xinjiang (36.4). The ADR per 100,000 due to SCD of the rural areas (42.1) was higher than that of the urban areas (37.3). However, there was no significant difference on the SRR between different areas or regions [Southern vs. Northern, SRR (95%CI): 1.13 (0.73–1.75); Rural vs. Urban, SRR (95%CI): 1.16 (0.75–1.81)] (Table 3).

The 11 sites were stratified by GDP to investigate the influence of economic development. Comparing to the areas of high economic development, incidence of SCD was significantly increased in places with poor economic development [SRR (95%CI): 1.55 (1.02–2.38)] (Table 3).

For cases of SCD, 4 ethnic groups were further stratified by underlying cardiovascular diseases (Table 4). In all ethnic groups, the most common underlying cardiovascular disease was coronary artery disease, followed by hypertension. These two diseases accounted for > 70% of all SCD cases. The percentages of SCD cases with a specific underlying cardiovascular disease were roughly consistent except for the Kazakh people, for whom the percentage of coronary heart disease was lower and the percentage of hypertension was higher compared with the other 3 groups. Of note, because of the lack of autopsy information, the underlying cardiovascular disease could not be identified in some SCD cases.

**Table 4:** Underlying cardiovascular diseases among the 4 ethnic groups with SCD, *n* (%)

	Han	Uyghur	Kazakh	Hui	P
Coronary artery disease	382 (60.2) ^a^	239 (52.4)	39 (47.0) ^a,b^	46 (65.7) ^b^	0.007
Hypertension	86 (13.5) ^a,c^	98 (21.5) ^c^	24 (28.9) ^a,b^	8 (11.4) ^b^	0
Cardiac valve disease	10 (1.6)	5 (1.1)	0 (0.0)	0 (0.0)	0.925
Ischemic cardiomyopathy	40 (6.3)	25 (5.5)	4 (4.8)	4 (5.7)	0.901
Dilated cardiomyopathy	13 (2.1)	13 (2.9)	1 (1.2)	0 (0.0)	0.680
Congenital heart disease	3 (0.5)	5 (1.1)	3 (3.6)	0 (0.0)	0.123
Pulmonary heart disease	14 (2.2)	4 (0.9)	0 (0.0)	1 (1.4)	0.356
Unknown cause	87 (13.7)	67 (14.7)	12 (14.5)	11 (15.7)	0.949

^a^Han compared with Kazakh, *P* < 0.05; ^b^ Hui compared with Kazakh, *P* < 0.05; ^c^ Han compared with Uyghur, *P* < 0.05

## Discussion

The peoples of Xinjiang comprise multiple ethnicities and religions, and differences in the incidence rates of cardiovascular diseases among them have been well documented [12, 13]. While the city of Kelamayi was selected for investigation in 2005 by Hua et al. [6], little is known of the epidemiological characteristics of SCD in other areas of Xinjiang. To rectify this lack of information, for the present study we sampled 11 sites within Xinjiang that were chosen with consideration for natural environment, economic development, ethnic distribution, and urban/rural differences. This is the first study of the epidemiological features of SCD among the ethnic groups Uyghur, Hui, and Kazakh, which were also compared with the Han ethnic group.

Overall, 1244 SCDs occurred among the 11 selected sites during the study period, with an estimated SCD incidence of 38.6 per 100,000 residents. While studies concerning SCD rates in China are limited, the overall rate of 38.6 per 100,000 residents that we report here is lower than the overall rate of 41.8 found by Hua et al. [6] while higher than the rate of 29.3 that that group reported specifically for the city of Kelamayi. A probable reason for these differences may lie with the demographic features of the study population, such as age, gender, ethnicity, and genetics [14]. Another reason may be a difference in the definition of SCD. Our present study excluded deaths that occurred more than 1 hour after the onset of acute symptoms, while the Hua et al. [6] study included deaths from 1 to 2 h after onset. Therefore, it is difficult to determine whether our results are truly distinct from the previous study. However, it is interesting to note that the estimated incidence of SCD in the current study was comparable with those reported in other countries of Asia such as Japan (37 per 100,000) and Tailand (38 per 100,000) [15, 16]. In addition, our findings confirmed the concept that the Asia population had a relatively lower risk of SCD compared with that in the western countries, which was possibly attributed to the different proportion of underlying diseases [17].

Our study showed that the men had a 1.75 fold risk of SCD compared to that of women. Although the mechanism was not analyzed in depth, differences in SCD incidence between the genders has been well reported in previous studies, with male-to-female ratios ranging from 1.1 to 4.6 [6, 15, 18–20]. It has been proposed that the lower incidence of SCD among women may be associated with estrogen, which is believed to have a protective effect on the cardiovascular system [21], as mediated by estrogen receptors, both directly and indirectly [22].

In addition, the higher rate of SCD among men may be attributed to the higher prevalence of tobacco smoking in men in China [23]. It was reported that for the year 2005, in China smoking was responsible for as many as 146,200 cardiovascular deaths [24]. As the Chinese government has engaged in programs that discourage the use of tobacco, the prevalence of smoking is expected to decline, and with it smoking-related deaths, including SCD.

Regarding ethnic groups in Xinjiang, in the present study the highest rate of SCD was detected in the Kazakh people, followed by the Uyghur, Han, and Hui. These variations may be ascribed to differences in cardiovascular disease among these peoples. In a national hypertension survey in China, among all 56 ethnic groups the Kazakh were among the top 5 in prevalence of hypertension, higher than the other 3 ethnic groups that were analyzed in the present study [25]. In the present study, we also found a higher proportion of hypertension among the Kazakh compared with the other 3 ethnic groups considered. This may be due to higher salt intake, which is inherited culturally from a formerly nomadic lifestyle [26]. Thus, the higher incidence of SCD in the Kazakh people may be related to the higher prevalence of hypertension. Studies have also shown that valvular heart diseases were more common in the Han and Kazakh compared with the Uyghur [13]. The prevalence of chronic heart failure was higher in the Kazakh than the Han or Uyghur [27], and the prevalence of peripheral arterial disease was higher in the Uyghur than the Kazakh [28].

The variations in SCD incidence between the 4 ethnic groups considered here may also be related to genetic polymorphisms. As the case control study of Abudoukelimu et al. [29] showed, differences in polymorphisms of the NUMB (protein numb homolog) gene were significantly associated with differences in coronary artery disease between the Han and Uyghur study populations.

We also stratified the 11 selected study sites according to 3 levels of economic development, and found that areas with the poorest level had the highest risk for SCD. This was consistent with Mensah et al.’s [30] study, which held that low economic status was a risk factor of cardiovascular death. Benefiting from continuous expansion and improvement of the medical security system in China, the gap in SCD incidence between high and low socioeconomic regions may become narrower.

Our study has several limitations. First, SCD cases were monitored for only 1 year, and therefore we could not analyze yearly variations in incidence. From this point of view, a further long-term cohort study is wanted. Secondly, the autopsy rate in China is low because of traditional views that regard autopsy as an insult or disrespect to the decedent [31]. In our study, no necropsies were performed. Lack of autopsy data prevented us from identifying the specific cause of death in 14.2% of all SCD cases. Thirdly, there are 47 ethnic groups residing in Xinjiang, and we only evaluated the SCD rates of 4 of these. Further investigation of the other ethnic groups should be implemented, to assess comprehensively the SCD rates in Xinjiang. Fourthly, sampling weights were not considered in the current study which might weaken the representativeness of the entire province.

## Conclusion

SCD remains an important public health issue in Xinjiang, China. Our understanding of the demographic differences on SCD in Xinjiang, China may improve the risk stratification and management to reduce the incidence and lethality of SCD. Underlying mechanisms of the demographic discrepancies are still needed to be investigated in the future.

## Abbreviations

GDP: Gross domestic product
SCD: Sudden cardiac death
